# Supplementary material for: Modeling glioblastoma heterogeneity as a dynamic network of cell states
Source: Mol Syst Biol. 2021 Sep 16;17(9):e10105. doi: 10.15252/msb.202010105 (PMC8444284; doi:10.15252/msb.202010105)
Supplement: Supplementary file 5 — Source Data for Figure 3 [file MSB-17-e10105-s001.zip › Figure3A_sourcedata/GSEA_3065/hallmarks_state6.GseaPreranked.1623416308702/HALLMARK_OXIDATIVE_PHOSPHORYLATION.html]

Details for gene set HALLMARK\_OXIDATIVE\_PHOSPHORYLATION[GSEA]

|  || Dataset | state6 |
| Phenotype | NoPhenotypeAvailable |
| Upregulated in class | na\_pos |
| GeneSet | HALLMARK\_OXIDATIVE\_PHOSPHORYLATION |
| Enrichment Score (ES) | 0.34707737 |
| Normalized Enrichment Score (NES) | 1.6099341 |
| Nominal p-value | 0.0 |
| FDR q-value | 0.03824241 |
| FWER p-Value | 0.088 |
Table: GSEA Results Summary

  

Fig 1: Enrichment plot: HALLMARK\_OXIDATIVE\_PHOSPHORYLATION      
 Profile of the Running ES Score & Positions of GeneSet Members on the Rank Ordered List

  

| PROBE | GENE SYMBOL | GENE\_TITLE | RANK IN GENE LIST | RANK METRIC SCORE | RUNNING ES | CORE ENRICHMENT || 1 | NDUFC1 |  |  | 37 | 0.255 | 0.0162 | Yes |
| 2 | BAX |  |  | 65 | 0.233 | 0.0318 | Yes |
| 3 | UQCR11 |  |  | 71 | 0.230 | 0.0498 | Yes |
| 4 | ATP5F1E |  |  | 88 | 0.219 | 0.0655 | Yes |
| 5 | SLC25A6 |  |  | 96 | 0.216 | 0.0820 | Yes |
| 6 | COX8A |  |  | 117 | 0.205 | 0.0962 | Yes |
| 7 | UQCRH |  |  | 208 | 0.174 | 0.0998 | Yes |
| 8 | ATP5MC2 |  |  | 213 | 0.171 | 0.1131 | Yes |
| 9 | COX6B1 |  |  | 216 | 0.170 | 0.1265 | Yes |
| 10 | SUCLG1 |  |  | 221 | 0.169 | 0.1397 | Yes |
| 11 | UQCR10 |  |  | 257 | 0.161 | 0.1486 | Yes |
| 12 | COX7A2L |  |  | 273 | 0.157 | 0.1594 | Yes |
| 13 | NDUFB2 |  |  | 312 | 0.149 | 0.1670 | Yes |
| 14 | COX7C |  |  | 325 | 0.147 | 0.1775 | Yes |
| 15 | COX6C |  |  | 340 | 0.145 | 0.1876 | Yes |
| 16 | POLR2F |  |  | 348 | 0.144 | 0.1984 | Yes |
| 17 | COX17 |  |  | 361 | 0.142 | 0.2084 | Yes |
| 18 | GPX4 |  |  | 365 | 0.141 | 0.2194 | Yes |
| 19 | ATP6V1F |  |  | 407 | 0.135 | 0.2255 | Yes |
| 20 | NDUFA3 |  |  | 414 | 0.134 | 0.2356 | Yes |
| 21 | ATP5ME |  |  | 424 | 0.132 | 0.2452 | Yes |
| 22 | ECI1 |  |  | 482 | 0.125 | 0.2486 | Yes |
| 23 | NDUFA5 |  |  | 492 | 0.124 | 0.2576 | Yes |
| 24 | COX5B |  |  | 543 | 0.119 | 0.2613 | Yes |
| 25 | UQCRC1 |  |  | 613 | 0.113 | 0.2624 | Yes |
| 26 | NDUFA6 |  |  | 730 | 0.103 | 0.2572 | Yes |
| 27 | COX4I1 |  |  | 742 | 0.103 | 0.2642 | Yes |
| 28 | COX7B |  |  | 800 | 0.098 | 0.2655 | Yes |
| 29 | MGST3 |  |  | 824 | 0.097 | 0.2706 | Yes |
| 30 | PHB2 |  |  | 828 | 0.096 | 0.2780 | Yes |
| 31 | ATP5MG |  |  | 853 | 0.094 | 0.2828 | Yes |
| 32 | ATP5PO |  |  | 856 | 0.094 | 0.2901 | Yes |
| 33 | NDUFB3 |  |  | 869 | 0.093 | 0.2962 | Yes |
| 34 | ATP5MC3 |  |  | 874 | 0.093 | 0.3032 | Yes |
| 35 | HTRA2 |  |  | 907 | 0.091 | 0.3068 | Yes |
| 36 | FDX1 |  |  | 916 | 0.090 | 0.3132 | Yes |
| 37 | PDHA1 |  |  | 977 | 0.087 | 0.3132 | Yes |
| 38 | NDUFA1 |  |  | 1014 | 0.085 | 0.3158 | Yes |
| 39 | ATP6V1C1 |  |  | 1172 | 0.077 | 0.3038 | Yes |
| 40 | ATP6V1E1 |  |  | 1173 | 0.077 | 0.3100 | Yes |
| 41 | GLUD1 |  |  | 1176 | 0.076 | 0.3159 | Yes |
| 42 | UQCRQ |  |  | 1209 | 0.075 | 0.3182 | Yes |
| 43 | TIMM13 |  |  | 1220 | 0.075 | 0.3231 | Yes |
| 44 | ISCU |  |  | 1241 | 0.074 | 0.3267 | Yes |
| 45 | TIMM8B |  |  | 1344 | 0.069 | 0.3205 | Yes |
| 46 | ATP5PF |  |  | 1379 | 0.068 | 0.3220 | Yes |
| 47 | NDUFA8 |  |  | 1410 | 0.067 | 0.3239 | Yes |
| 48 | COX7A2 |  |  | 1411 | 0.067 | 0.3293 | Yes |
| 49 | ALDH6A1 |  |  | 1436 | 0.066 | 0.3319 | Yes |
| 50 | HADHB |  |  | 1451 | 0.066 | 0.3356 | Yes |
| 51 | NDUFA2 |  |  | 1458 | 0.066 | 0.3402 | Yes |
| 52 | ETFB |  |  | 1467 | 0.066 | 0.3445 | Yes |
| 53 | NDUFS3 |  |  | 1504 | 0.064 | 0.3455 | Yes |
| 54 | ACAA2 |  |  | 1540 | 0.062 | 0.3464 | Yes |
| 55 | CYB5A |  |  | 1578 | 0.061 | 0.3471 | Yes |
| 56 | FH |  |  | 1622 | 0.060 | 0.3469 | No |
| 57 | MAOB |  |  | 1701 | 0.057 | 0.3424 | No |
| 58 | ATP5MF |  |  | 1794 | 0.054 | 0.3360 | No |
| 59 | SLC25A3 |  |  | 1835 | 0.052 | 0.3356 | No |
| 60 | ATP5PD |  |  | 1847 | 0.052 | 0.3385 | No |
| 61 | ACAT1 |  |  | 1870 | 0.052 | 0.3401 | No |
| 62 | MRPL11 |  |  | 1932 | 0.050 | 0.3370 | No |
| 63 | ACADVL |  |  | 1940 | 0.049 | 0.3402 | No |
| 64 | ISCA1 |  |  | 2007 | 0.048 | 0.3364 | No |
| 65 | MRPS15 |  |  | 2065 | 0.046 | 0.3334 | No |
| 66 | ATP6V1D |  |  | 2082 | 0.045 | 0.3352 | No |
| 67 | NDUFB7 |  |  | 2093 | 0.045 | 0.3377 | No |
| 68 | MRPL15 |  |  | 2130 | 0.044 | 0.3371 | No |
| 69 | GPI |  |  | 2151 | 0.043 | 0.3382 | No |
| 70 | UQCRB |  |  | 2152 | 0.043 | 0.3417 | No |
| 71 | SURF1 |  |  | 2163 | 0.043 | 0.3440 | No |
| 72 | CYB5R3 |  |  | 2172 | 0.043 | 0.3465 | No |
| 73 | ATP5F1C |  |  | 2266 | 0.039 | 0.3389 | No |
| 74 | ACAA1 |  |  | 2308 | 0.038 | 0.3372 | No |
| 75 | MFN2 |  |  | 2449 | 0.035 | 0.3237 | No |
| 76 | MRPS12 |  |  | 2501 | 0.033 | 0.3205 | No |
| 77 | IDH1 |  |  | 2508 | 0.033 | 0.3224 | No |
| 78 | VDAC2 |  |  | 2516 | 0.033 | 0.3243 | No |
| 79 | NDUFS4 |  |  | 2545 | 0.032 | 0.3236 | No |
| 80 | LRPPRC |  |  | 2574 | 0.032 | 0.3229 | No |
| 81 | TIMM10 |  |  | 2632 | 0.030 | 0.3187 | No |
| 82 | MPC1 |  |  | 2724 | 0.028 | 0.3104 | No |
| 83 | PDP1 |  |  | 2766 | 0.027 | 0.3079 | No |
| 84 | GOT2 |  |  | 2783 | 0.027 | 0.3082 | No |
| 85 | SUCLA2 |  |  | 2844 | 0.026 | 0.3033 | No |
| 86 | AIFM1 |  |  | 2865 | 0.025 | 0.3030 | No |
| 87 | OPA1 |  |  | 2883 | 0.024 | 0.3030 | No |
| 88 | UQCRC2 |  |  | 2924 | 0.024 | 0.3002 | No |
| 89 | SLC25A11 |  |  | 2937 | 0.023 | 0.3007 | No |
| 90 | ATP6V1G1 |  |  | 2997 | 0.022 | 0.2957 | No |
| 91 | NDUFB4 |  |  | 3026 | 0.021 | 0.2941 | No |
| 92 | ATP5F1D |  |  | 3028 | 0.021 | 0.2957 | No |
| 93 | NDUFB1 |  |  | 3119 | 0.020 | 0.2869 | No |
| 94 | ECH1 |  |  | 3187 | 0.018 | 0.2805 | No |
| 95 | RHOT1 |  |  | 3279 | 0.016 | 0.2712 | No |
| 96 | DECR1 |  |  | 3363 | 0.014 | 0.2627 | No |
| 97 | ACADM |  |  | 3368 | 0.014 | 0.2634 | No |
| 98 | MDH1 |  |  | 3403 | 0.013 | 0.2605 | No |
| 99 | NDUFA4 |  |  | 3449 | 0.012 | 0.2562 | No |
| 100 | DLAT |  |  | 3553 | 0.010 | 0.2451 | No |
| 101 | MRPL34 |  |  | 3608 | 0.009 | 0.2395 | No |
| 102 | IDH3G |  |  | 3728 | 0.006 | 0.2262 | No |
| 103 | HSD17B10 |  |  | 3779 | 0.005 | 0.2208 | No |
| 104 | TOMM22 |  |  | 3787 | 0.005 | 0.2204 | No |
| 105 | NDUFAB1 |  |  | 3836 | 0.004 | 0.2151 | No |
| 106 | CS |  |  | 3864 | 0.003 | 0.2123 | No |
| 107 | SDHC |  |  | 3913 | 0.002 | 0.2069 | No |
| 108 | CASP7 |  |  | 3926 | 0.002 | 0.2056 | No |
| 109 | NDUFV2 |  |  | 3939 | 0.002 | 0.2044 | No |
| 110 | SLC25A5 |  |  | 4174 | -0.003 | 0.1774 | No |
| 111 | MRPS11 |  |  | 4363 | -0.006 | 0.1560 | No |
| 112 | NDUFA7 |  |  | 4364 | -0.006 | 0.1565 | No |
| 113 | RHOT2 |  |  | 4371 | -0.006 | 0.1564 | No |
| 114 | PHYH |  |  | 4372 | -0.006 | 0.1569 | No |
| 115 | BDH2 |  |  | 4405 | -0.007 | 0.1537 | No |
| 116 | UQCRFS1 |  |  | 4466 | -0.008 | 0.1474 | No |
| 117 | MRPS22 |  |  | 4495 | -0.009 | 0.1448 | No |
| 118 | HCCS |  |  | 4496 | -0.009 | 0.1455 | No |
| 119 | ABCB7 |  |  | 4497 | -0.009 | 0.1462 | No |
| 120 | NQO2 |  |  | 4551 | -0.010 | 0.1409 | No |
| 121 | LDHB |  |  | 4572 | -0.010 | 0.1394 | No |
| 122 | MTX2 |  |  | 4838 | -0.015 | 0.1098 | No |
| 123 | OAT |  |  | 4839 | -0.015 | 0.1111 | No |
| 124 | NDUFB5 |  |  | 4853 | -0.016 | 0.1108 | No |
| 125 | AFG3L2 |  |  | 4863 | -0.016 | 0.1110 | No |
| 126 | ATP5F1A |  |  | 4972 | -0.018 | 0.0999 | No |
| 127 | SDHD |  |  | 5023 | -0.019 | 0.0956 | No |
| 128 | NDUFS6 |  |  | 5037 | -0.019 | 0.0956 | No |
| 129 | NDUFB8 |  |  | 5238 | -0.023 | 0.0743 | No |
| 130 | MRPS30 |  |  | 5327 | -0.025 | 0.0660 | No |
| 131 | IDH3B |  |  | 5335 | -0.025 | 0.0672 | No |
| 132 | OXA1L |  |  | 5395 | -0.026 | 0.0625 | No |
| 133 | TOMM70 |  |  | 5472 | -0.028 | 0.0559 | No |
| 134 | VDAC1 |  |  | 5528 | -0.029 | 0.0519 | No |
| 135 | NDUFS1 |  |  | 5530 | -0.029 | 0.0541 | No |
| 136 | PRDX3 |  |  | 5634 | -0.031 | 0.0447 | No |
| 137 | ECHS1 |  |  | 5643 | -0.031 | 0.0463 | No |
| 138 | COX11 |  |  | 5647 | -0.032 | 0.0485 | No |
| 139 | IDH3A |  |  | 5673 | -0.032 | 0.0481 | No |
| 140 | ATP6V0B |  |  | 5714 | -0.033 | 0.0461 | No |
| 141 | ALAS1 |  |  | 5763 | -0.034 | 0.0433 | No |
| 142 | NDUFS2 |  |  | 5981 | -0.038 | 0.0211 | No |
| 143 | MRPL35 |  |  | 6027 | -0.039 | 0.0190 | No |
| 144 | CYCS |  |  | 6090 | -0.040 | 0.0150 | No |
| 145 | SUPV3L1 |  |  | 6117 | -0.041 | 0.0153 | No |
| 146 | ATP5PB |  |  | 6130 | -0.041 | 0.0172 | No |
| 147 | DLD |  |  | 6247 | -0.044 | 0.0072 | No |
| 148 | ATP5F1B |  |  | 6367 | -0.046 | -0.0029 | No |
| 149 | CYC1 |  |  | 6383 | -0.047 | -0.0008 | No |
| 150 | ATP6V1H |  |  | 6385 | -0.047 | 0.0028 | No |
| 151 | COX15 |  |  | 6392 | -0.047 | 0.0059 | No |
| 152 | ATP5MC1 |  |  | 6395 | -0.047 | 0.0094 | No |
| 153 | MDH2 |  |  | 6431 | -0.048 | 0.0092 | No |
| 154 | SDHB |  |  | 6839 | -0.060 | -0.0332 | No |
| 155 | IDH2 |  |  | 6985 | -0.064 | -0.0449 | No |
| 156 | ACO2 |  |  | 7057 | -0.066 | -0.0478 | No |
| 157 | COX6A1 |  |  | 7089 | -0.067 | -0.0459 | No |
| 158 | SDHA |  |  | 7125 | -0.069 | -0.0445 | No |
| 159 | HADHA |  |  | 7134 | -0.069 | -0.0399 | No |
| 160 | VDAC3 |  |  | 7150 | -0.069 | -0.0360 | No |
| 161 | PDHX |  |  | 7155 | -0.070 | -0.0309 | No |
| 162 | COX5A |  |  | 7157 | -0.070 | -0.0254 | No |
| 163 | NDUFS7 |  |  | 7178 | -0.070 | -0.0221 | No |
| 164 | NDUFS8 |  |  | 7241 | -0.072 | -0.0234 | No |
| 165 | IMMT |  |  | 7374 | -0.077 | -0.0326 | No |
| 166 | TIMM17A |  |  | 7403 | -0.078 | -0.0295 | No |
| 167 | TIMM9 |  |  | 7437 | -0.079 | -0.0270 | No |
| 168 | PDHB |  |  | 7466 | -0.080 | -0.0238 | No |
| 169 | SLC25A4 |  |  | 7519 | -0.082 | -0.0232 | No |
| 170 | TIMM50 |  |  | 7614 | -0.087 | -0.0272 | No |
| 171 | OGDH |  |  | 7799 | -0.096 | -0.0409 | No |
| 172 | ATP6V0E1 |  |  | 7802 | -0.096 | -0.0334 | No |
| 173 | NDUFV1 |  |  | 7842 | -0.098 | -0.0300 | No |
| 174 | FXN |  |  | 7877 | -0.100 | -0.0259 | No |
| 175 | NDUFA9 |  |  | 7893 | -0.101 | -0.0195 | No |
| 176 | GRPEL1 |  |  | 7920 | -0.102 | -0.0143 | No |
| 177 | PMPCA |  |  | 7923 | -0.102 | -0.0063 | No |
| 178 | ATP6AP1 |  |  | 7998 | -0.107 | -0.0063 | No |
| 179 | ATP1B1 |  |  | 8091 | -0.113 | -0.0079 | No |
| 180 | HSPA9 |  |  | 8097 | -0.113 | 0.0007 | No |
| 181 | ETFA |  |  | 8139 | -0.117 | 0.0053 | No |
| 182 | LDHA |  |  | 8261 | -0.127 | 0.0015 | No |
| 183 | NNT |  |  | 8335 | -0.136 | 0.0040 | No |
| 184 | NDUFB6 |  |  | 8469 | -0.155 | 0.0010 | No |
| 185 | NDUFC2 |  |  | 8681 | -0.228 | -0.0051 | No |
| 186 | POR |  |  | 8682 | -0.228 | 0.0132 | No |
Table: GSEA details [plain text format]

  

Fig 2: HALLMARK\_OXIDATIVE\_PHOSPHORYLATION: Random ES distribution      
 Gene set null distribution of ES for **HALLMARK\_OXIDATIVE\_PHOSPHORYLATION**
